# *Shigella* in Africa: New Insights From the Vaccine Impact on Diarrhea in Africa (VIDA) Study

**DOI:** 10.1093/cid/ciac969

**Published:** 2023-04-19

**Authors:** Irene N Kasumba, Henry Badji, Helen Powell, M Jahangir Hossain, Richard Omore, Samba O Sow, Jennifer R Verani, James A Platts-Mills, Marc-Alain Widdowson, Syed M A Zaman, Jennifer Jones, Sunil Sen, Jasnehta Permala-Booth, Shamima Nasrin, Anna Roose, Dilruba Nasrin, John Benjamin Ochieng, Jane Juma, Sanogo Doh, Joquina Chiquita M Jones, Martin Antonio, Alex O Awuor, Ciara E Sugerman, Nora Watson, Christopher Focht, Jie Liu, Eric Houpt, Karen L Kotloff, Sharon M Tennant

**Affiliations:** Center for Vaccine Development and Global Health, University of Maryland School of Medicine, Baltimore, Maryland; Department of Medicine, University of Maryland School of Medicine, Baltimore, Maryland; Medical Research Council Unit, The Gambia at the London School of Hygiene and Tropical Medicine, The Gambia, Banjul; Center for Vaccine Development and Global Health, University of Maryland School of Medicine, Baltimore, Maryland; Department of Pediatrics, University of Maryland School of Medicine, Baltimore, Maryland; Medical Research Council Unit, The Gambia at the London School of Hygiene and Tropical Medicine, The Gambia, Banjul; Center for Global Health Research, Kenya Medical Research Institute, Kenya, Kisumu; Centre pour le Développement des Vaccins du Mali, Mali, Bamako; Division of Global Health Protection, US Centers for Disease Control and Prevention, Nairobi, Kenya; Division of Infectious Diseases and International Health, Department of Medicine, University of Virginia, Charlottesville, Virginia; Division of Global Health Protection, US Centers for Disease Control and Prevention, Nairobi, Kenya; Medical Research Council Unit, The Gambia at the London School of Hygiene and Tropical Medicine, The Gambia, Banjul; Center for Vaccine Development and Global Health, University of Maryland School of Medicine, Baltimore, Maryland; Department of Medicine, University of Maryland School of Medicine, Baltimore, Maryland; Center for Vaccine Development and Global Health, University of Maryland School of Medicine, Baltimore, Maryland; Department of Medicine, University of Maryland School of Medicine, Baltimore, Maryland; Center for Vaccine Development and Global Health, University of Maryland School of Medicine, Baltimore, Maryland; Department of Medicine, University of Maryland School of Medicine, Baltimore, Maryland; Center for Vaccine Development and Global Health, University of Maryland School of Medicine, Baltimore, Maryland; Department of Medicine, University of Maryland School of Medicine, Baltimore, Maryland; Center for Vaccine Development and Global Health, University of Maryland School of Medicine, Baltimore, Maryland; Department of Pediatrics, University of Maryland School of Medicine, Baltimore, Maryland; Center for Vaccine Development and Global Health, University of Maryland School of Medicine, Baltimore, Maryland; Department of Medicine, University of Maryland School of Medicine, Baltimore, Maryland; Center for Global Health Research, Kenya Medical Research Institute, Kenya, Kisumu; Center for Global Health Research, Kenya Medical Research Institute, Kenya, Kisumu; Centre pour le Développement des Vaccins du Mali, Mali, Bamako; Medical Research Council Unit, The Gambia at the London School of Hygiene and Tropical Medicine, The Gambia, Banjul; Medical Research Council Unit, The Gambia at the London School of Hygiene and Tropical Medicine, The Gambia, Banjul; Center for Global Health Research, Kenya Medical Research Institute, Kenya, Kisumu; Division of Foodborne, Waterborne, and Environmental Diseases, US Centers for Disease Control and Prevention, Atlanta, Georgia; The Emmes Company, Rockville, Maryland; The Emmes Company, Rockville, Maryland; Division of Infectious Diseases and International Health, Department of Medicine, University of Virginia, Charlottesville, Virginia; School of Public Health, Qingdao University, China; Division of Infectious Diseases and International Health, Department of Medicine, University of Virginia, Charlottesville, Virginia; Center for Vaccine Development and Global Health, University of Maryland School of Medicine, Baltimore, Maryland; Department of Medicine, University of Maryland School of Medicine, Baltimore, Maryland; Department of Pediatrics, University of Maryland School of Medicine, Baltimore, Maryland; Center for Vaccine Development and Global Health, University of Maryland School of Medicine, Baltimore, Maryland; Department of Medicine, University of Maryland School of Medicine, Baltimore, Maryland

**Keywords:** Shigella, diarrhea, Africa, children, dysentery

## Abstract

**Background:**

We evaluated the burden of *Shigella* spp from children aged 0–59 months with medically attended moderate-to-severe diarrhea and matched controls at sites in Mali, The Gambia, and Kenya participating in the Vaccine Impact on Diarrhea in Africa (VIDA) study from 2015 to 2018.

**Methods:**

*Shigella* spp were identified using coprocultures and serotyping in addition to quantitative polymerase chain reaction (qPCR). Episode-specific attributable fractions (AFe) for *Shigella* were calculated using *Shigella* DNA quantity; cases with AFe ≥0.5 were considered to have shigellosis.

**Results:**

The prevalence of *Shigella* was determined to be 359 of 4840 (7.4%) cases and 83 of 6213 (1.3%) controls by culture, and 1641 of 4836 (33.9%) cases and 1084 of 4846 (22.4%) controls by qPCR (cycle threshold <35); shigellosis was higher in The Gambia (30.8%) than in Mali (9.3%) and Kenya (18.7%). Bloody diarrhea attributed to *Shigella* was more common in 24- to 59-month-old children (50.1%) than 0- to 11-month-old infants (39.5%). The *Shigella flexneri* serogroup predominated among cases (67.6% of isolates), followed by *Shigella sonnei* (18.2%), *Shigella boydii* (11.8%), and *Shigella dysenteriae* (2.3%). The most frequent *S*. *flexneri* serotypes were 2a (40.6%), 1b (18.8%), 6 (17.5%), 3a (9.0%), and 4a (5.1%). Drug-specific resistance among 353 (98.3%) *Shigella* cases with AMR data was as follows: trimethoprim-sulfamethoxazole (94.9%), ampicillin (48.4%), nalidixic acid (1.7%), ceftriaxone (0.3%), azithromycin (0.3%), and ciprofloxacin (0.0%).

**Conclusions:**

A high prevalence of shigellosis continues in sub-Saharan Africa. Strains are highly resistant to commonly used antibiotics while remaining susceptible to ciprofloxacin, ceftriaxone, and azithromycin.

## Teaser

We evaluated the burden of *Shigella* spp from children 0–59 months with medically attended moderate-to-severe diarrhea and matched controls at sites in Mali, The Gambia, and Kenya participating in the Vaccine Impact on Diarrhea in Africa study from 2015 to 2018.

In the Global Enteric Multicenter Study (GEMS), *Shigella* was the second most common cause of medically attended moderate-to-severe diarrhea (MSD) in children aged 12–23 months at 7 sites in sub-Saharan Africa and South Asia and the leading pathogen among children 24–59 months of age [1]. While incidence varied, infection was common at all sites. Moreover, the attributable incidence more than doubled when the samples were reanalyzed using quantitative polymerase chain reaction (qPCR) compared to culture [2]. These findings, subsequently confirmed in other populations of children from low- and middle-income countries (LMICs) [3, 4], even in studies that only evaluated children with watery diarrhea [5], created a new awareness of the importance of the burden of shigellosis and invigorated efforts to develop a vaccine [6]. As clinical development of new vaccines proceeds, decision makers must examine the public health value of vaccine introduction. Because ongoing social and economic trends and a variety of environmental and host factors can impact the epidemiology of shigellosis, up-to-date information is needed on disease burden, including both direct outcomes (acute diarrhea and dysentery) and indirect effects such as stunting, hospitalization, mortality, and healthcare costs, in the context of increasing antimicrobial resistance (AMR). Finally, continued monitoring of the serotype distribution of strains will help to inform the characteristics of vaccines that are appropriate for the settings where the need is greatest.

The Vaccine Impact on Diarrhea in Africa (VIDA) study was conducted in 3 sub-Saharan African countries that participated in GEMS (The Gambia, Mali, and Kenya) and utilized comparable methodology to determine the incidence, etiology, and adverse clinical consequences of MSD following rotavirus vaccine introduction. Using qPCR, we found that *Shigella* spp had the highest attributable incidence of any pathogen in 12- to 23-month-old and 24- to 59-month-old children at all 3 sites [Kotloff et al., unpublished data]. Herein we aim to define the prevalence, seasonality, and clinical features as well as the serotype diversity and antibiotic susceptibility of *Shigella* spp identified in stool samples of children enrolled in the VIDA study. Furthermore, we compared serotype distribution and antimicrobial susceptibility of *Shigella* isolates collected during VIDA (2015–2018) to GEMS (1 December 2007 to 3 March 2011) and the GEMS-1A follow-up study (31 October 2011 to 14 November 2012).

## METHODS

### Study Participants and Enrollment

The methods and main results from VIDA have been detailed elsewhere [7]. In brief, between May 2015 and May 2018 in The Gambia, May 2015 to May 2018 in Mali, and July 2015 to July 2018 in Kenya, all children 0–59 months of age living within an ongoing demographic surveillance system (DSS) and who presented at a sentinel health center (SHC) serving the DSS population with diarrhea (≥3 episodes of abnormally loose stools within the previous 24 hours with onset within the previous 7 days) were assessed for the presence of MSD, defined as diarrhea plus 1 or more of the following: sunken eyes (more than usual per the caretaker), blood in stool, decreased skin turgor, requiring intravenous fluids, or recommended for hospitalization. We aimed to enroll 8–9 MSD cases per fortnight into each of 3 age strata (0–11 months, 12–23 months, and 24–59 months) over the 36 month study period at each site to manage workload. For each case of MSD, we enrolled 1–3 controls (depending on case recruitment rate) within 14 days of the index case who were matched to the case by age, sex, and residence in the same or a nearby village and who had no history of diarrhea within the preceding 7 days. Controls were randomly selected from the DSS database.

### Collection of Stool Samples

Cases and controls donated a whole stool specimen (minimum 4 g). Cases donated the stool within 12 hours of entering the SHC. If a case was to receive antibiotics at the SHC, 2 rectal swabs were also collected for identification of pathogens by culture. Controls provided the whole stool at their enrollment visit, which took place in the home. An aliquot of the whole stool sample from cases and controls, as well as the rectal swabs, were placed in buffered glycerol saline and Cary-Blair transport media immediately (rectal swabs) or within 6 hours of passage (whole stool aliquots) and transported together to the laboratory at 2°C–8°C in a cooler box within 18 hours of receipt.

### Detection and Identification of *Shigella* Strains in Stool Samples

*Shigella* spp were identified in cases and controls at each site using conventional bacteriological culture techniques as previously described [8, 9]. *Shigella* isolates were then shipped to the Center for Vaccine Development and Global Health (CVD), Baltimore, Maryland, for serotyping by slide agglutination test using commercial antisera (Denka Seiken, Japan). In addition, stools from all cases of MSD and the first matched control for each case were tested for the presence of the *ipaH* gene to identify *Shigella/*enteroinvasive *Escherichia coli* by qPCR using TaqMan Array Cards at each site, as previously described herein termed *Shigella* [2, 10].

### Antibiotic Susceptibility Testing

Antibiotic susceptibility testing was performed at the CVD using the Kirby-Bauer disk diffusion method according to the Clinical and Laboratory Standards Institute procedures and guidelines (M100, 31st edition). Antibiotic disks (BD BBL Sensi-Disc, Becton Dickinson) used were ampicillin (10 µg), azithromycin (15 µg), ceftriaxone (30 µg), ciprofloxacin (5 µg), nalidixic acid (30 µg), and trimethoprim-sulfamethoxazole (25 µg).

### Seasonality

We obtained rainfall and temperature data from the government of The Gambia Annual Climate report 2018 and the Kenya Meteorological Department, Kisumu International Airport station for The Gambia and Kenya, respectively. Modeled monthly estimates of rainfall and temperature were obtained for Mali [11]. To examine the monthly seasonal trends of *Shigella*, we applied a site, age group, and calendar month–specific weight consisting of the total number of children from the DSS meeting criteria for MSD who presented to an SHC divided by the total number of MSD cases enrolled. The total number of attributable and nonattributable *Shigella* MSD cases (defined below) for a given month and site was calculated as the sum over all instances of that month within the study period and over all age groups.

### Data Analysis

An episode-specific attributable fraction (AFe) for each MSD episode that was positive for *Shigella* by PCR (cycle threshold [Ct] <35) was calculated as described elsewhere [7]; cases with an AFe ≥0.5 were considered attributable (shigellosis), which translates to a pathogen-specific Ct value associated with a 2-fold or higher odds of being an MSD case after controlling for site, age group, and presence of other pathogens. MSD cases that were qPCR positive for *Shigella* but had an AFe <0.5 were considered not attributable to *Shigella* and therefore designated as nonshigellosis. Therefore, the criteria for a PCR-positive case to be considered attributable takes into consideration the pathogen load (Ct value) in cases and controls, age, and site, and adjusts for all other pathogens. Clinical symptoms among shigellosis cases were compared to those of other pathogens to determine if there was a particular constellation of symptoms that could define shigellosis.

All analyses involving *Shigella* serotype or assessment of AMR used *Shigella* MSD episodes in which the isolate was identified by classical microbiological methods. Those with multiple serotypes of *Shigella flexneri* were removed to allow for comparisons. We examined the predilection among the 4 serogroups (*S. flexneri*, *Shigella sonnei*, *Shigella boydii*, and *Shigella dysenteriae*) detected by culture for causing bloody (persistent or acute), acute watery, or persistent watery diarrhea. Temporal trends in serogroup/serotype distribution were examined from the beginning of GEMS (December 2007) through the end of VIDA (July 2018). We examined the distribution of antibiotic susceptibility of *Shigella* isolated from cases by serogroup and evaluated annual trends in antimicrobial susceptibility patterns among *Shigella* isolates from cases of MSD collected during GEMS, GEMS-1A, and VIDA studies.

Comparisons were made using χ^2^ tests, or Fisher test in the event that <5 observations were expected, and Wilcoxon rank-sum tests. A *P* value <.05 was considered statistically significant. All analyses were conducted using R version 4.2.0 or SAS version 9.4 software.

### Ethical Review

This project was approved by the institutional review boards of the University of Maryland, Baltimore (HP-00062472); the US Centers for Disease Control and Prevention (CDC), Atlanta, Georgia (reliance agreement, CDC protocol number 6729); The Gambia government/Medical Research Council/Gambia at the London School of Hygiene and Tropical Medicine (1409); the Comité d'Ethique de la Faculté de Médecine, de Pharmacie, et d'Odonto-Stomatologie, Bamako, Mali (no number); and the Kenya Medical Research Institute Scientific and Ethics Review Unit in Siaya County, Kenya (SSE 2996). Written informed consent was obtained from the parent or primary caretaker of each child who met eligibility criteria before any research activities were performed.

## RESULTS

### Detection of *Shigella* in Stool

**Figure 1.**
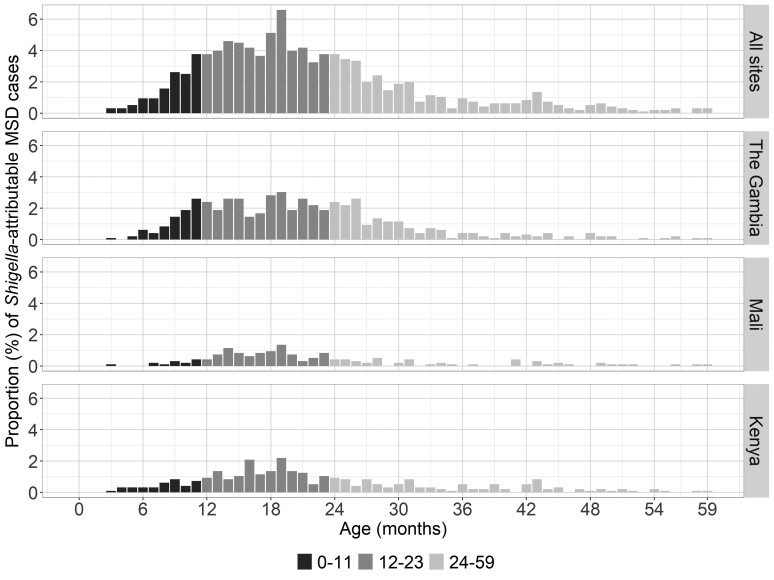
The age distribution among children with moderate-to-severe diarrhea (MSD) attributable to *Shigella*/enteroinvasive *Escherichia coli* by quantitative polymerase chain reaction at each site.

Both culture and qPCR results are shown in Table 1. qPCR was more sensitive at detecting *Shigella* DNA than the conventional stool culture method was at detecting whole organisms (Supplementary Table 1). The proportion of MSD cases in which *Shigella* was detected was 38.1% by qPCR versus 6.9% by culture in The Gambia, 24.0% versus 0.5% in Mali, and 21.8% versus 4.4% in Kenya. We attributed 957 of 4836 (19.8%) MSD cases to *Shigella* spp (defined as AFe ≥0.5 by qPCR) (Table 1). The number of attributable cases varied across sites, with the highest observed in The Gambia (30.8%), followed by Kenya (18.7%) and Mali (9.3%). We found a similar trend in the age distribution of shigellosis cases across sites; shigellosis peaked (6.6%) at 19 months of age (Figure 1).

**Table 1.** Positivity of *Shigella* Among Stools of Vaccine Impact on Diarrhea in Africa (VIDA) Study Cases and Controls by Both Culture and Quantitative Polymerase Chain Reaction (qPCR), Including the Number of qPCR-Positive Moderate-to-Severe Cases That Are Attributable to *Shigella*

Assay	All Sites		The Gambia		Mali		Kenya	
	Cases (n = 4840)	Controls (n = 6213)	Cases (n = 1678)	Controls (n = 2138)	Cases (n = 1608)	Controls (n = 1980)	Cases (n = 1554)	Controls (n = 2095)
Stool culture, No. (%)	359 (7.4)	83 (1.3)	217 (12.9)	47 (2.2)	12 (0.7)	6 (0.3)	130 (8.4)	30 (1.4)
qPCR tested, No.^a^	4836^b^	4836	1677	1677	1608	1608	1551	1551
ȃNo. (%) positive (Ct <35)	1641 (33.9)	1084 (22.4)	770 (45.9)	507 (30.2)	438 (27.2)	334 (20.8)	433 (27.9)	243 (15.7)
ȃNo. (%) attributable to *Shigella* (shigellosis, AFe ≥0.5)	957 (19.8)	…	517 (30.8)	…	150 (9.3)	…	290 (18.7)	…

Abbreviations: AFe, episode-specific attributable fraction; Ct, cycle threshold; qPCR, quantitative polymerase chain reaction.

Only the first matched control was tested by qPCR.

Two case samples were never tested and 2 cases had to be removed due to incorrect IDs.

### Clinical Syndromes of Shigellosis

**Figure 2.**
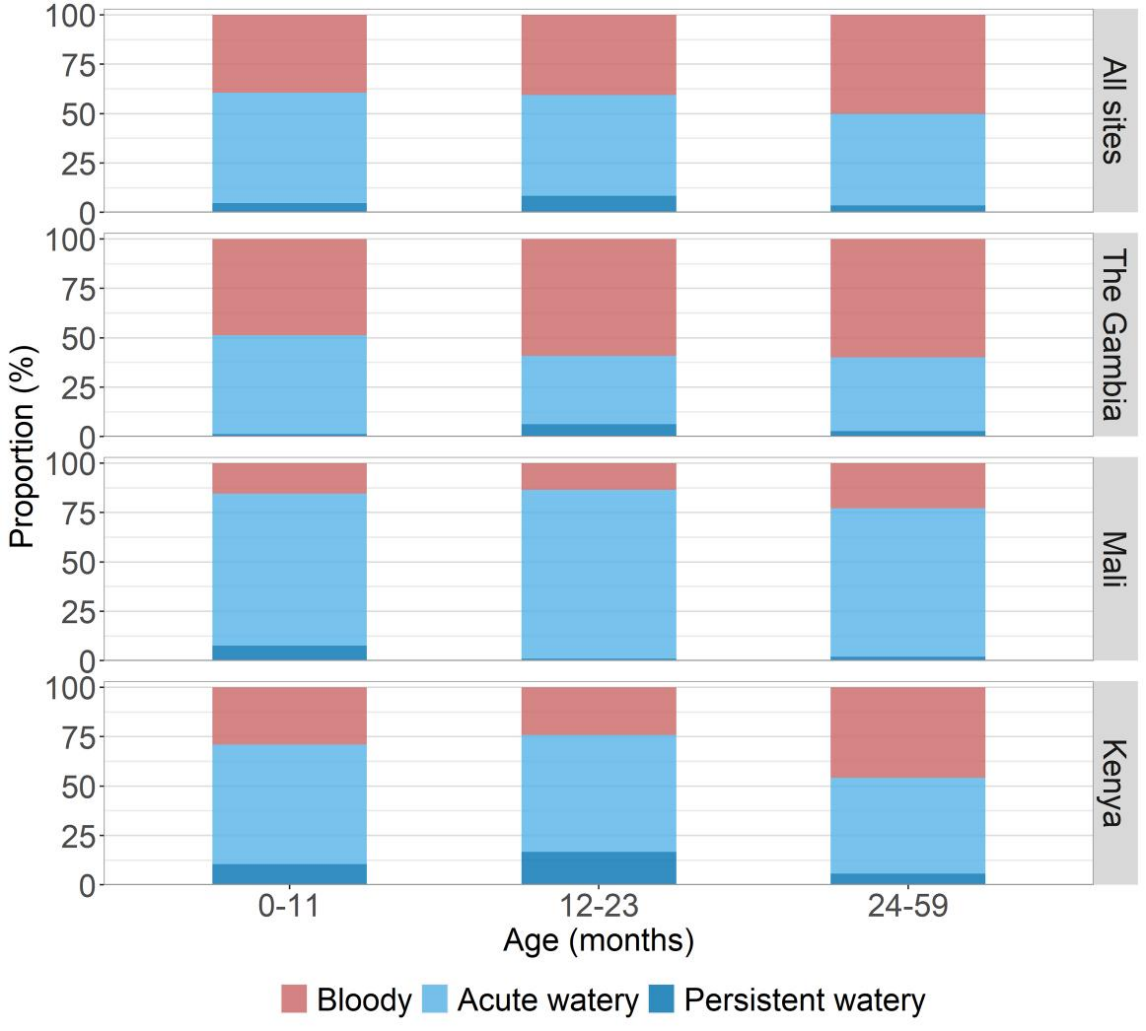
Clinical syndromes among shigellosis cases. Proportions of attributable *Shigella*/enteroinvasive *Escherichia coli* cases with bloody, acute watery, and persistent watery diarrhea are shown by age group and Vaccine Impact on Diarrhea in Africa (VIDA) study sites.

There were both age- as well as site-specific differences in the clinical syndromes experienced by the 957 children with attributable shigellosis (Figure 2 and Supplementary Table 2; age group vs clinical syndrome, *P* = .006; site vs clinical syndrome, *P* < .001). Overall, most children with attributable shigellosis presented with acute or persistent watery diarrhea (0–11 months: 78/129 [60.5%]; 12–23 months: 293/493 [59.4%]; 24–59 months: 167/335 [49.9%], all sites), with the exception of the 2 older age groups in The Gambia, whose predominant presentation was dysentery. The proportion of children presenting with bloody stools was higher among the older age groups (0–11 months: 51/129 [39.5%]; 12–23 months: 200/493 [40.6%]; 24–59 months: 168/335 [50.1%], all sites). Moreover, there were site-specific differences, with attributable shigellosis cases in Mali (0–11 months: 2/13 [15.4%]; 12–23 months: 12/89 [13.5%]; 24–59 months: 11/48 [22.9%]) being less likely to present with bloody diarrhea than Kenya (0–11 months: 11/38 [28.9%]; 12–23 months: 35/145 [24.1%]; 24–59 months: 49/107 [45.8%]) or The Gambia (0–11 months: 38/78 [48.7%]; 12–23 months: 153/259 [59.1%]; 24–59 months: 108/180 [60.0%]). In terms of clinical symptoms associated with shigellosis (other than blood in stool), only vomiting showed a statistically significant difference (less for *Shigella* than for other pathogens) for all age groups (0–11 months: 18/48 [37.5%] for *Shigella* vs 497/733 [67.8%] for other pathogens, *P* < .0001; 12–23 months: 58/153 [37.9%] vs 299/465 [64.3%], *P* < .0001; 24–59 months: 42/108 [38.9%] vs 149/265 [56.2%], *P* = .0029) (Supplementary Tables 3–5).

### Seasonal Distribution of Shigellosis at VIDA Sites

**Figure 3.**
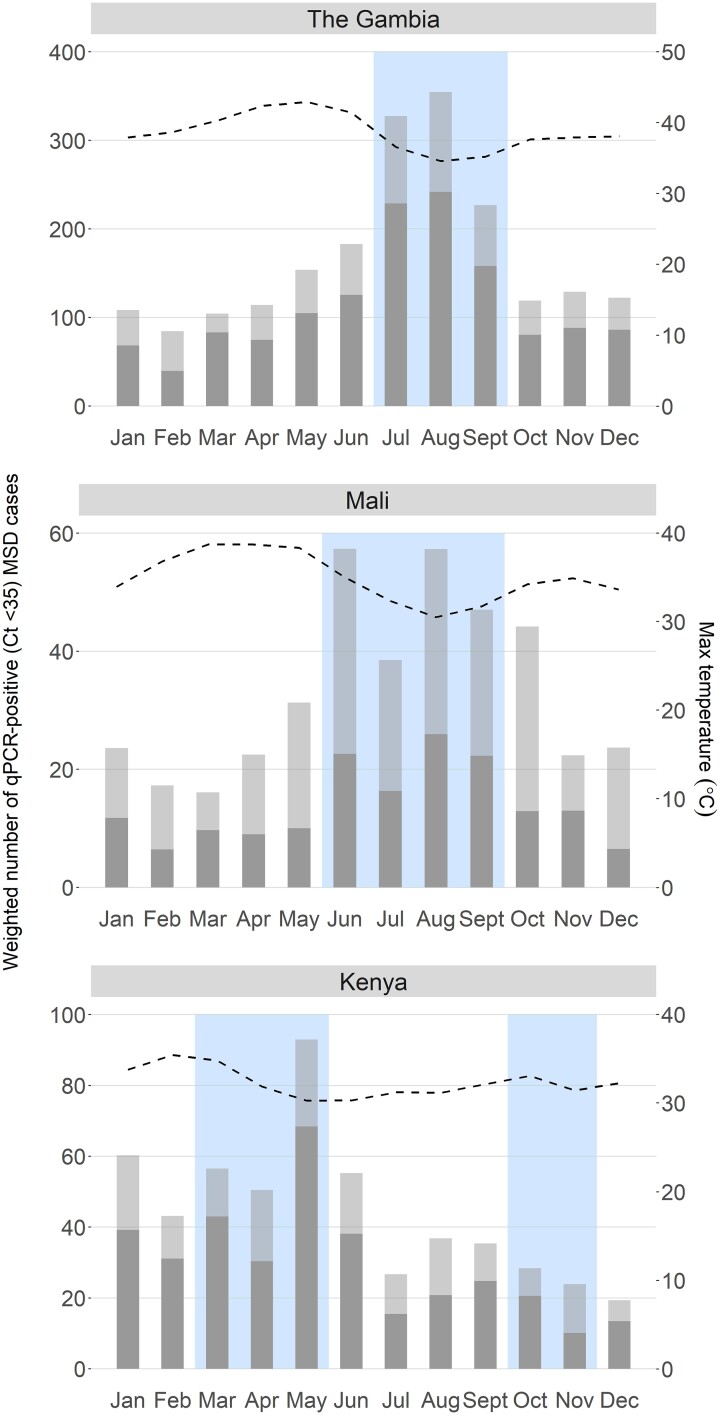
Seasonality of attributable and nonattributable *Shigella* moderate-to-severe diarrhea (MSD) cases in The Gambia, Mali, and Kenya during the Vaccine Impact on Diarrhea in Africa (VIDA) study. Data are shown as the number of attributable (episode-specific attributable fraction [AFe] ≥0.5, dark gray bar) and nonattributable cases (cycle threshold <35 and AFe <0.5, light gray bar) per month weighted by the number of children with MSD who could have been enrolled by site (as opposed to the capped enrollment number). Dotted line represents maximum monthly temperature and shaded box represent months with rainfall above the study period average. Abbreviations: Ct, cycle threshold; MSD, moderate-to-severe diarrhea; qPCR, quantitative polymerase chain reaction.

We determined the relationship between monthly rainfall, monthly maximum temperature, and shigellosis among children with MSD during VIDA using qPCR data. Figure 3 shows that peak detection of attributable *Shigella* occurred during periods of greater than average rainfall during this time period. The rainy season occurred between July to September in The Gambia and between June to September in Mali. At the Kenya site, 46% of all cases of shigellosis occurred during each of the 2 rainy seasons: a primary peak during the long rains of March to May (33.5%) and a lower a secondary peak during the shorter rainy season in October to November (12.5%).

### Proportion of *Shigella* Serogroups and the Distribution of Clinical Syndromes by Serogroup

The proportion of *Shigella* serogroups and *S*. *flexneri* serotypes identified by stool culture during VIDA is shown in Table 2. *Shigella flexneri* was the most abundant serogroup detected in stools of cases (234/347 [67.6%]) and controls (43/82 [52.4%]), followed by *S. sonnei*, *S. boydii*, and *S. dysenteriae* (Table 2). *Shigella flexneri* serotypes 1b, 2a, 3a, and 6 were the most common serotypes. We found a significantly higher proportion of children infected with *S. flexneri* (141/234 [60.3%]) had bloody stools compared to infection with *S. sonnei* (28/63 [44.4%]) (*P* = .0352; Supplementary Figure 1 and Supplementary Table 6). There were no other significant differences in clinical symptoms between *S. flexneri* and *S. sonnei*–infected children (Supplementary Table 6). There were site-specific differences in the proportion of each serogroup isolated from children with bloody versus watery diarrhea. In The Gambia, 96 of 207 (46.4%) of the MSD cases presented as bloody diarrhea in which *S. flexneri* was isolated. In contrast, in Kenya, only 42 of 128 (32.8%) of the MSD cases presented with bloody diarrhea and had *S. flexneri* (Supplementary Table 7).

**Table 2.** Distribution of *Shigella* Serogroups and Serotypes Identified From Stool Culture Isolates During Vaccine Impact on Diarrhea in Africa (VIDA) Study Sites From Cases and Controls From Whom a Single *Shigella* Serotype Was Isolated^a^

*Shigella* Serogroup/Serotype	No. (%) of Cases and Controls With Each Serogroup/Serotype							
	All Sites		The Gambia		Mali		Kenya	
	Cases (n = 347)	Controls (n = 82)	Cases (n = 207)	Controls (n = 46)	Cases (n = 12)	Controls (n = 6)	Cases (n = 128)	Controls (n = 30)
*S*. *boydii*	41 (11.8)	12 (14.6)	30 (14.5)	8 (17.4)	1 (8.3)	0 (0)	10 (7.8)	4 (13.3)
*S*. *sonnei*	63 (18.2)	16 (19.5)	24 (11.6)	10 (21.7)	4 (33.3)	0 (0)	35 (27.3)	6 (20.0)
*S*. *dysenteriae*	8 (2.3)	11 (13.4)	6 (2.9)	5 (10.9)	0 (0)	1 (16.7)	2 (1.6)	5 (16.7)
*S*. *flexneri*	234 (67.6)	43 (52.4)	146 (70.5)	23 (50.0)	7 (58.3)	5 (83.3)	81 (63.3)	15 (50.0)
ȃ1a	1 (0.4)	0 (0)	1 (0.5)	0 (0)	0 (0)	0 (0)	0 (0)	0 (0)
ȃ1b	44 (18.8)	5 (11.6)	37 (17.9)	4 (17.9)	0 (0)	0 (0)	7 (8.6)	1 (6.7)
ȃ1d	2 (0.9)	0 (0)	2 (1.0)	0 (0)	0 (0)	0 (0)	0 (0)	0 (0)
ȃ2a	95 (40.6)	7 (16.3)	52 (25.1)	3 (13.0)	6 (85.7)	1 (20.0)	37 (45.7)	3 (20.0)
ȃ2b	5 (2.1)	4 (9.3)	5 (2.4)	3 (13.0)	0 (0)	1 (20.0)	0 (0)	0 (0)
ȃ3a	21 (9.0)	7 (16.3)	8 (3.9)	2 (8.7)	0 (0)	0 (0)	13 (16.0)	5 (33.3)
ȃ3b	3 (1.3)	0 (0)	3 (1.4)	0 (0)	0 (0)	0 (0)	0 (0)	0 (0)
ȃ4a	12 (5.1)	4 (9.3)	9 (4.3)	2 (8.7)	0 (0)	0 (0)	3 (3.7)	2 (13.3)
ȃ4b	2 (0.9)	0 (0)	1 (0.5)	0 (0)	0 (0)	0 (0)	1 (1.2)	0 (0)
ȃ4c	0 (0)	0 (0)	0 (0)	0 (0)	0 (0)	0 (0)	0 (0)	0 (0)
ȃ6	41 (17.5)	14 (32.6)	26 (12.6)	9 (19.6)	0 (0)	1 (20.0)	15 (18.5)	4 (26.7)
ȃ7a	2 (0.9)	1 (2.3)	1 (0.5)	0 (0)	0 (0)	1 (20.0)	1 (1.2)	0 (0)
ȃX	4 (1.7)	1 (2.3)	0 (0)	0 (0)	1 (14.3)	1 (20.0)	3 (3.7)	0 (0)
ȃY	2 (0.9)	0 (0)	1 (0.5)	0 (0)	0 (0)	0 (0)	1 (1.2)	0 (0)
*Shigella* spp	0 (0)	0 (0)	1 (0.5)	0 (0)	0 (0)	0 (0)	0 (0)	0 (0)

Children who had multiple serogroups isolated from their stool (Gambia: 10 cases and 1 control; Kenya: 1 case) were excluded, as were children who had multiple *S. flexneri* types (Kenya: 1 case).

**Figure 4.**
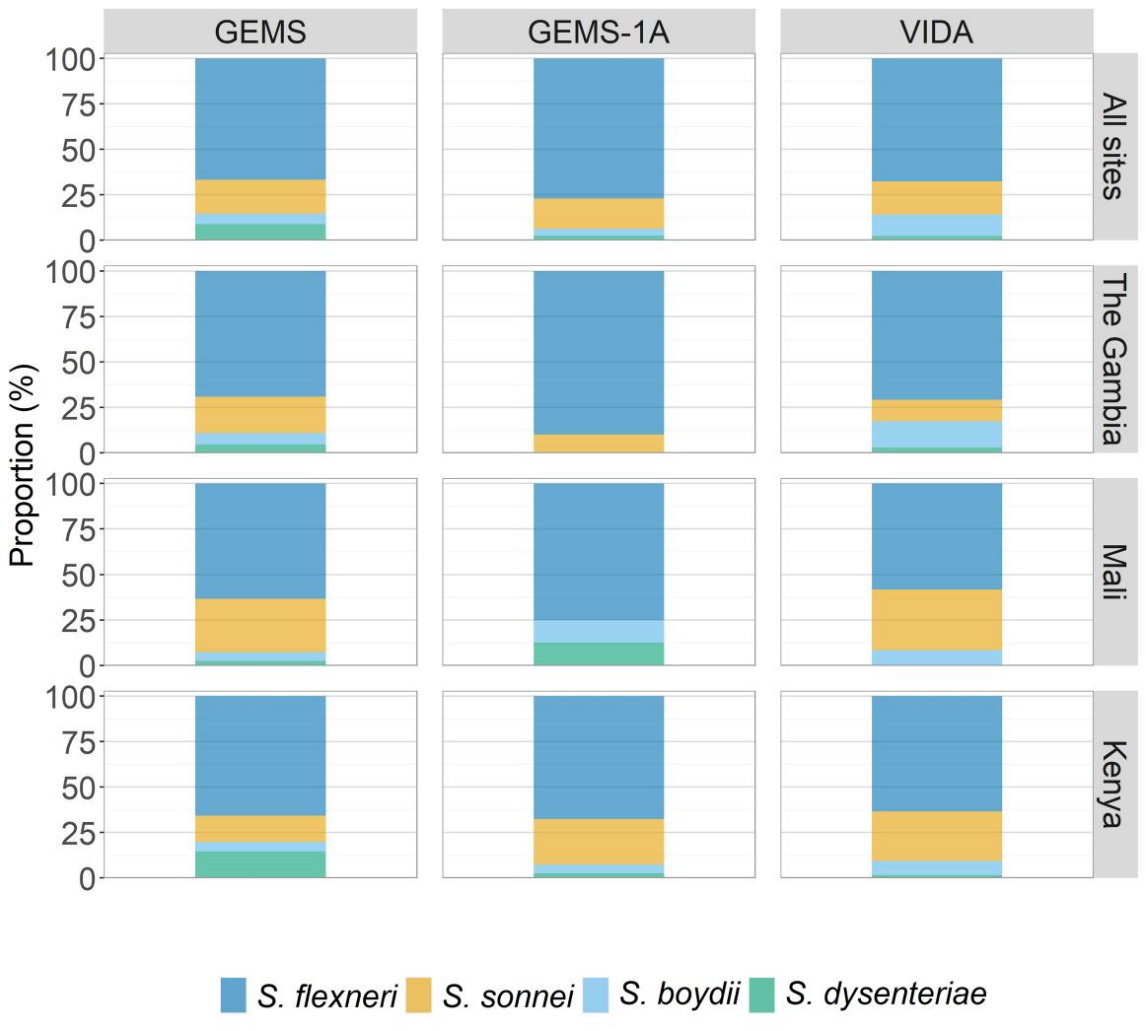
Proportion of *Shigella* serogroups from stools of cases of moderate-to-severe diarrhea at the 3 Vaccine Impact on Diarrhea in Africa (VIDA) study sites. The frequency of *Shigella*-positive cases during 3 study periods (Global Enteric Multicenter Study [GEMS], GEMS-1A, and VIDA) is shown for each of the 3 study sites in sub-Saharan Africa.

The distribution of *Shigella* serogroups and serotypes remained relatively constant over time, although there were some site-specific fluctuations (Figure 4 and Supplementary Figure 2). Most notably, *S. dysenteriae* was observed in Kenya during GEMS (25/282 [8.9%]) but decreased to 2 of 78 [2.6%] during GEMS-1A and 8 of 347 [2.3%] during VIDA.

### Antimicrobial Susceptibility of *Shigella*

**Figure 5.**
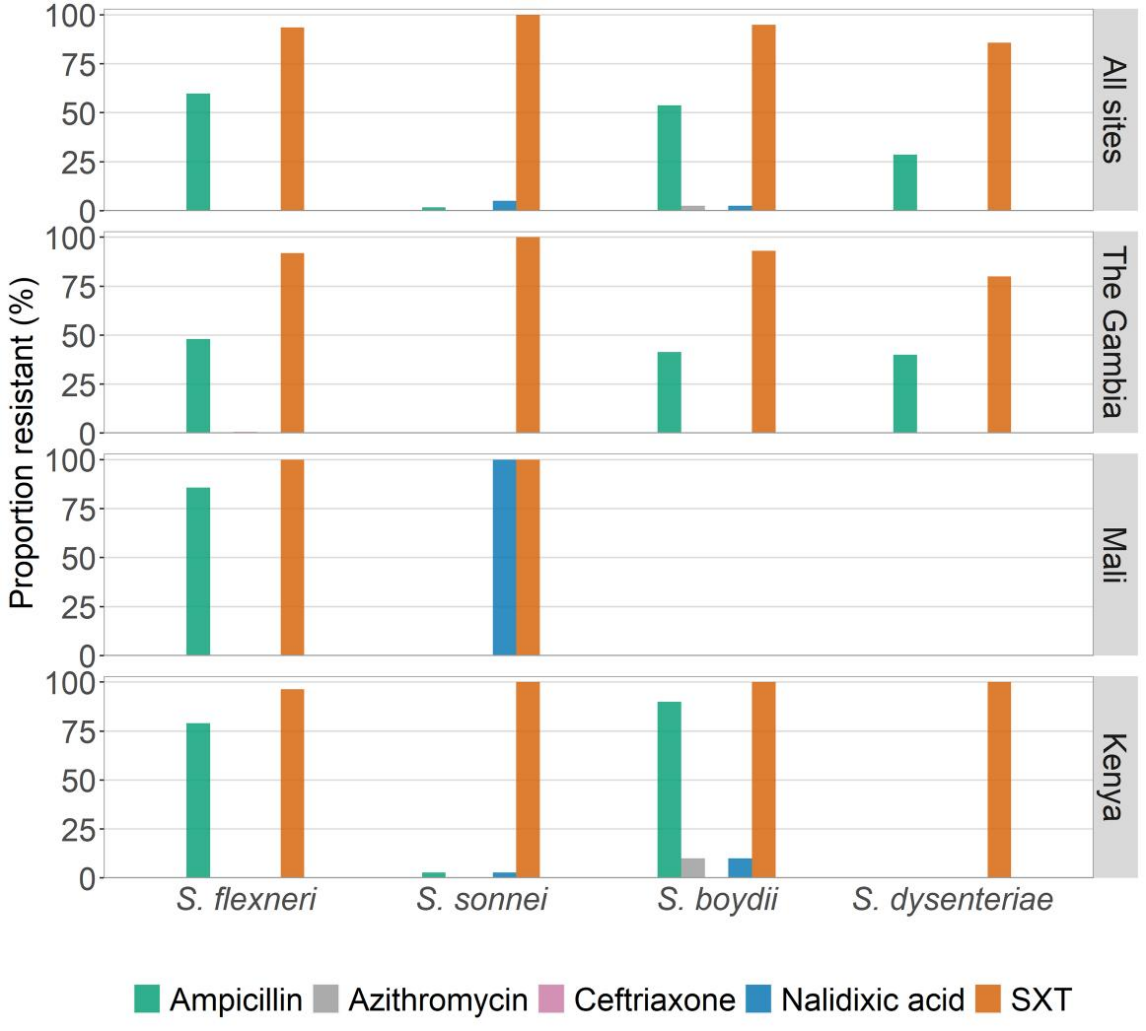
Proportion of strains in each *Shigella* serogroup that exhibited antimicrobial resistance, by site. Abbreviation: SXT, trimethoprim-sulfamethoxazole.

**Figure 6.**
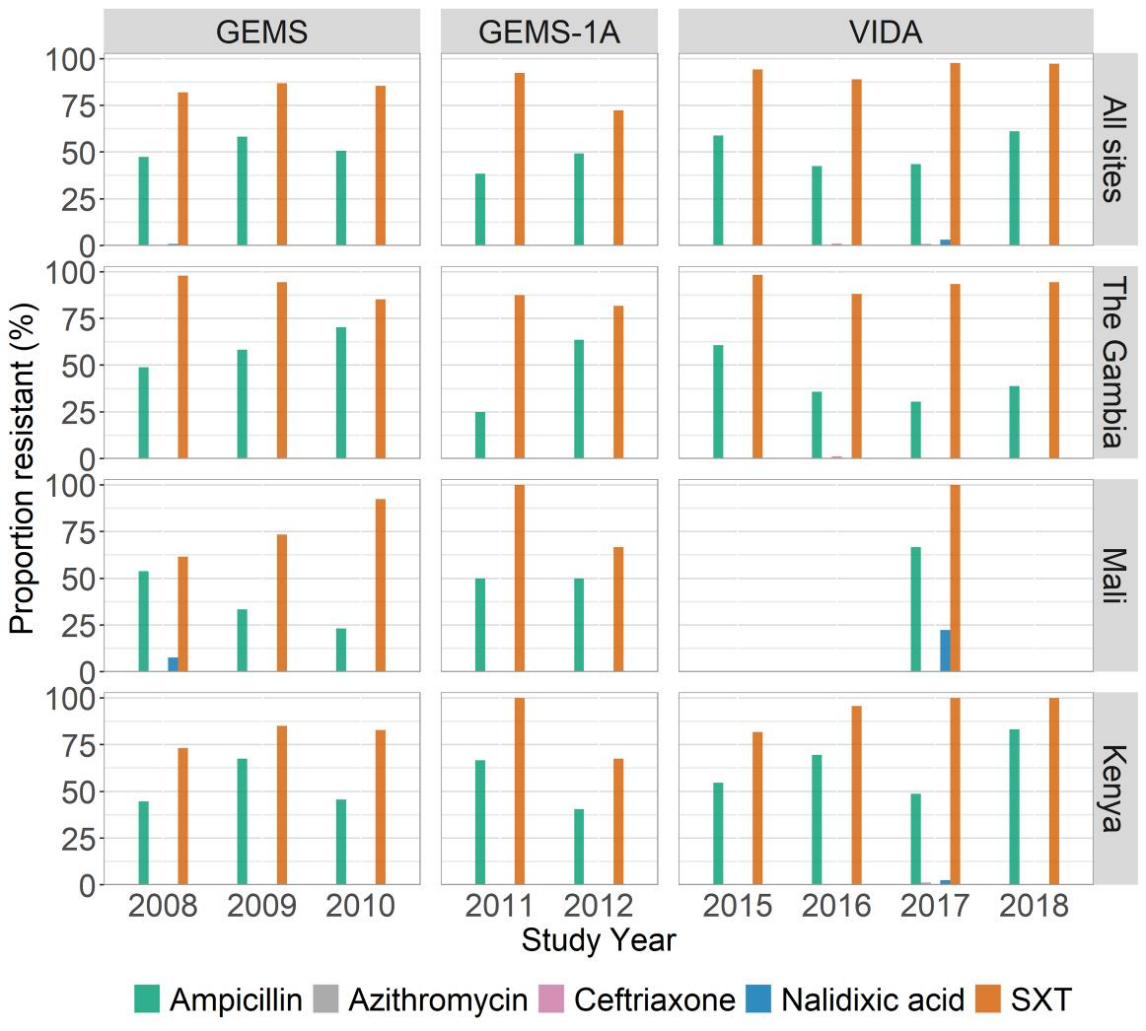
Annual proportions of isolates that displayed antimicrobial resistance, by site. Abbreviations: GEMS, Global Enteric Multicenter Study; SXT, trimethoprim-sulfamethoxazole; VIDA, Vaccine Impact on Diarrhea in Africa.

The highest prevalence of AMR among *Shigella* isolates during VIDA was against SXT (94.9% among cases) (Table 3). Next was resistance to ampicillin (48.4% among cases). All isolates were susceptible to ciprofloxacin. Low levels of resistance were observed for nalidixic acid (1.7% among cases), ceftriaxone (0.3% among cases), and azithromycin (0.3% among cases). We observed similar susceptibility patterns for *S. flexneri* and *S. boydii*, while *S. sonnei* (9/60 [15.0%]) was less resistant to ampicillin compared to the other serogroups (146/234 [62.4%] for *S. flexneri*, 22/39 [56.4%] for *S. boydii*, and 2/7 [28.6%] for *S. dysenteriae*; Figure 5). Notably, the proportion of *Shigella* isolates demonstrating resistance to each antibiotic tested remained similar over the 11-year period of observation, with the exception of resistance to ceftriaxone which arose in The Gambia in 2016 (Figure 6).

**Table 3.** Antibiotic Resistance of *Shigella* Isolates That Were Evaluated During the Vaccine Impact on Diarrhea in Africa (VIDA) Study

Antibiotics Tested^b^	No. (%) of Isolates From Cases and Controls^a^ That Demonstrated Resistance							
	All		The Gambia		Mali		Kenya	
	Cases (n = 353)	Controls (n = 81)	Cases (n = 214)	Controls (n = 46)	Cases (n = 9)	Controls (n = 5)	Cases (n = 130)	Controls (n = 30)
Ampicillin	171 (48.4)	37 (45.7)	89 (41.6)	14 (30.4)	6 (66.7)	5 (100.0)	76 (58.5)	18 (60.0)
Azithromycin	1 (0.3)	1 (1.2)	0 (0)	1 (2.2)	0 (0)	0 (0)	1 (0.8)	0 (0)
Ceftriaxone	1 (0.3)	1 (1.2)	1 (0.5)	1 (2.2)	0 (0)	0 (0)	0 (0)	0 (0)
Nalidixic acid	6 (1.7)	1 (1.2)	2 (0.9)	1 (2.2)	2 (22.2)	0 (0)	2 (1.5)	0 (0)
Trimethoprim-sulfamethoxazole	335 (94.9)	76 (93.8)	199 (93.0)	42 (91.3)	9 (100.0)	4 (80.0)	127 (97.7)	30 (100.0)

Including from children who had multiple serotypes isolated.

All isolates were susceptible to ciprofloxacin.

## DISCUSSION

The key findings of our study are that *Shigella* is common in sub-Saharan Africa, and the distribution of serotypes is stable over time. We observed low resistance to antibiotics that are recommended for empiric treatment of bloody diarrhea; however, the majority of *Shigella* cases presented as watery diarrhea.

We determined that peak shigellosis and carriage (qPCR positive among cases; Ct <35) of *Shigella* coincided with peak rainfall and below-average temperature at all 3 sites in sub-Saharan Africa. However, while we and other studies report an association between rainfall, temperature, and shigellosis, studies at other sites have shown no consistent association between rainfall, relative humidity, air pressure, or temperature and shigellosis [12–18]. These disparate findings suggest that the effect of environmental factors associated with shigellosis should be defined locally at each site before any *Shigella* intervention study or implementation of prevention efforts.

Our determination that *Shigella* generally manifests as watery diarrhea has important public health implications, since dysentery, not watery diarrhea, is an indication for antibiotics [19]. Recent evidence suggests that toddlers with MSD associated with *Shigella* who were treated with antibiotics had improved linear growth [20] and reduced duration of diarrhea [21]. Whether those with shigellosis who present with watery diarrhea would benefit from treatment is unknown; exploratory studies are ongoing [22]. Expanding indications for treatment of shigellosis to those with watery diarrhea would result in substantial increases in antibiotic exposure and the risk of AMR and must be considered with risk-to-benefit in mind.

There were age- and site-specific differences in the frequency of dysentery as the presenting finding in children with shigellosis. The proportion of attributable shigellosis presenting with bloody stools was highest in the oldest age group at each site. Our data suggest that the site-specific differences in clinical presentation are due to the serogroups circulating in these 3 sites. *S. flexneri* is considered an endemic species that predominates in low-resource settings [23, 24]; economic development results in a reduction in *S. flexneri*, and the relative proportion of *S. sonnei* thereby increases [19]. The finding that among our 3 study sites, The Gambia has the poorest health indicators (eg, maternal education, frequency of stunting) and the lowest proportion of *S. sonnei* is consistent with this observation [25]. Our data also showed that a significantly higher proportion of children infected with *S. flexneri* had bloody stools than children infected with *S. sonnei*. The association of *S. flexneri* with bloody diarrhea has previously been reported, although no statistical analyses were performed [26, 27]. Accordingly, the predominance of bloody diarrhea as the presenting manifestation at The Gambian site in our study could be explained by the high prevalence of *S. flexneri*. Our data suggest that cases of diarrhea due to *S. sonnei* may be missed as this serotype is more likely to elicit watery than bloody diarrhea, which could lead to transmission if the need for rigorous hygiene is not recognized.

When we examined the 3 studies, we found that the landscape of *Shigella* serogroups recovered from VIDA stools was similar to isolates from GEMS stools, with few shifts in serotypes during the 10 years since the start of GEMS. Based on animal studies, a multivalent vaccine containing *S*. *sonnei* and *S*. *flexneri* serotypes 2a, 3a, and 6 O polysaccharides should provide direct and indirect protection against *S. sonnei* and all *S. flexneri* serotypes except 7a [28]. Data from GEMS showed that a multivalent vaccine targeting these 4 serotypes would theoretically provide direct protection against 66% of *Shigella* in The Gambia, 61% in Mali, and 40% in Kenya (low direct protection during GEMS due to the increased prevalence of *S. dysenteriae* in Kenya during this time period). In VIDA, we observed that direct protection would be theoretically achieved against 53% of *Shigella* cases in The Gambia, 83% in Mali, and 78% in Kenya. Indirect protection based on cross-protection by *S. flexneri* serotypes would be achieved against 82% of *Shigella* cases in The Gambia, 92% in Mali, and 90% in Kenya.

Our results indicated that there was no major shift in AMR during the 10 years since the start of our GEMS studies through VIDA. The highest levels were observed for SXT and ampicillin, while ciprofloxacin, azithromycin, and ceftriaxone resistance were minimal. This mirrors the prescribing patterns for both watery diarrhea and dysentery at these sites, which is dominated by SXT [20]. High resistance to these drugs has also been reported by studies from elsewhere in sub-Saharan Africa, including in earlier studies from Kenya [26] and more recently from the Central African Republic [29], Mozambique [30], and Ethiopia [31, 32]. Because our findings will prompt efforts at the sites to administer antibiotics to which the local *Shigella* are susceptible, such as ciprofloxacin, increasing rates of ciprofloxacin resistance are likely to ensue. Notably, *S. sonnei* was susceptible to ampicillin, a finding that has been reported elsewhere [26, 29, 33, 34]. The reason for the higher susceptibility of *S. sonnei* to (penicillin-derived) ampicillin is not clear and remains to be determined. Collectively, our data and those of previous studies strongly suggest that the use of cotrimoxazole, chloramphenicol, and ampicillin (and other penicillin-derived antibiotics) for the treatment of bloody diarrhea in sub-Saharan Africa should be discontinued unless local susceptibility data indicate otherwise, and clinicians should be encouraged to prescribe ciprofloxacin as the first choice and azithromycin, cefixime, and ceftriaxone as second choices as per World Health Organization (WHO) guidelines [19].

The major limitations of this study were the gaps in the data (2012–2014), incomplete years, and that qPCR did not provide serogroup or serotype identity. Notably Mali had the lowest sensitivity in the detection of *Shigella* by culture compared to The Gambia and Kenya. Mali also showed poor sensitivity for detection of *Salmonella* by culture [35]. The reduced detection by culture may have been due to antibiotic usage [36] or inadequate specimen transport (temperature and/or time to the laboratory), or culture techniques despite concerted efforts to enhance quality control at each of the sites.

In conclusion, we have found that *Shigella* continues to be an important diarrheal pathogen. Our findings raise 2 issues with important implications for antibiotic-prescribing practices for shigellosis. For one, even though many children experienced dysentery, more than 50% presented with watery diarrhea and therefore should not receive antibiotics according to WHO guidelines, which limit antibiotic use to children with bloody diarrhea. The potential benefits of treating children in LMICs with shigellosis who present with watery diarrhea, according to underlying health parameters, must be understood in relation to the risks of AMR. Second, while current prescription practices at our sites have preserved the susceptibility of *Shigella* to newer-generation antibiotics (ciprofloxacin and azithromycin) and parenteral agents (ceftriaxone), this has occurred at the expense of prescribing ineffective therapy. A switch to newer antibiotics such as ciprofloxacin would be judicious but will likely promote further AMR. There remains hope that both of these conundrums can be addressed by preventing shigellosis with a safe and effective *Shigella* vaccine.

## Supplementary Data

Supplementary materials are available at *Clinical Infectious Diseases* online. Consisting of data provided by the authors to benefit the reader, the posted materials are not copyedited and are the sole responsibility of the authors, so questions or comments should be addressed to the corresponding author.

## Supplementary Material

ciac969_Supplementary_Data

## Notes

***Acknowledgments.*** The authors thank the families who participated in these studies; the clinical and field staff for their exceptional hard work and dedication; and the physicians, administration, and health officials at every site who generously provided facilities and support for the conduct of the study. The authors are also grateful to Catherine Johnson at the Emmes Company, LLC, for expert data management and reporting. Special thanks go to Carl Kirkwood, Duncan Steele, and Anita Zaidi at the Bill & Melinda Gates Foundation for helpful oversight; Kathy Neuzil for thoughtful suggestions; and to the following members of our International Scientific Advisory Committee for providing insightful comments and guidance: Janet Wittes (Chair), George Armah, John Clemens, Christopher Duggan, Stephane Helleringer, Ali Mokdad, James Nataro, and Halvor Sommerfelt.

***Disclaimer*.** The funding organizations had no role in the design, collection, analysis, or interpretation of data, or in the writing of the manuscript. The findings and conclusions in this report are those of the authors and do not necessarily represent the official position of the Kenya Medical Research Institute, the University of Maryland, the US Centers for Disease Control and Prevention (CDC), or any of the collaborating partners in this project.

***Financial support.*** This work was supported by the Bill & Melinda Gates Foundation (grant numbers OPP1111236 and OPP1116751).

***Supplement sponsorship*.** This supplement was sponsored by the Bill & Melinda Gates Foundation. This study is based on research funded in part by grants from the Bill & Melinda Gates Foundation (OPP1111236/OPP1116751).
