# Supplementary material for: Shigella in Africa: New Insights From the Vaccine Impact on Diarrhea in Africa (VIDA) Study
Source: Clin Infect Dis. 2023 Apr 19;76(Suppl 1):S66–76. doi: 10.1093/cid/ciac969 (PMC10116563; doi:10.1093/cid/ciac969)
Supplement: ciac969_Supplementary_Data [file ciac969_supplementary_data.zip › Supplementary Figure 1.pdf]

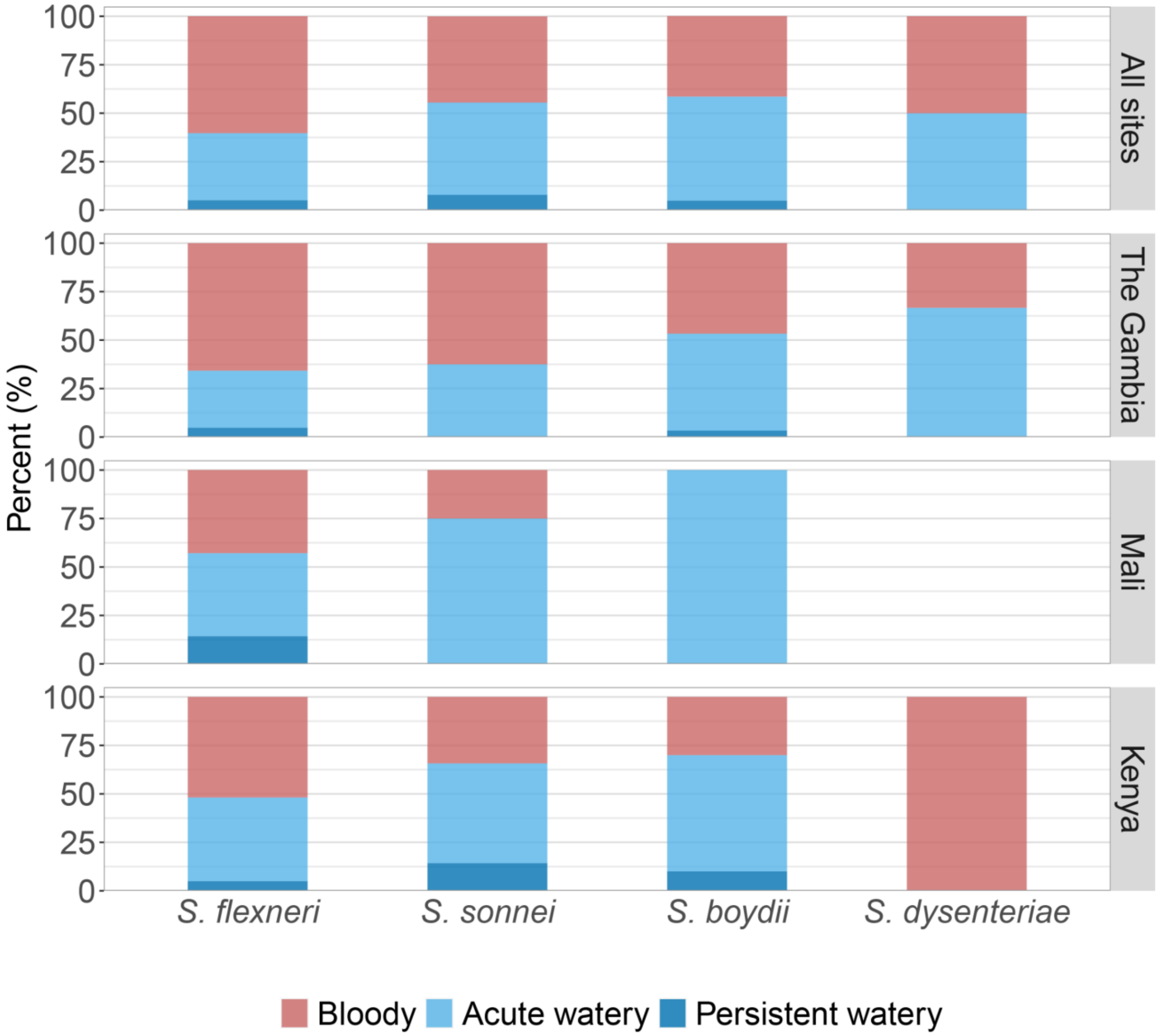

Supplementary Figure 1. Clinical syndromes of *Shigella* positive VIDA cases of MSD. Percent of *Shigella* positive cases with bloody, acute watery or persistent watery diarrhea is shown by serogroup across the three study sites.
