# Supplementary material for: Shigella in Africa: New Insights From the Vaccine Impact on Diarrhea in Africa (VIDA) Study
Source: Clin Infect Dis. 2023 Apr 19;76(Suppl 1):S66–76. doi: 10.1093/cid/ciac969 (PMC10116563; doi:10.1093/cid/ciac969)
Supplement: ciac969_Supplementary_Data [file ciac969_supplementary_data.zip › Supplementary table_1.pdf]

Supplementary Table 1. Sensitivity and specificity of stool culture versus qPCR by site in VIDA.

		qPCR											
		The Gambia				Mali				Kenya			
		Cases		Controls		Cases		Controls		Cases		Controls	
		**Positive**[a]	**Negative**	**Positive**	**Negative**	**Positive**	**Negative**	**Positive**	**Negative**	**Positive**	**Negative**	**Positive**	**Negative**
**Stool Culture (denominator)**	**Positive**[b]	204/217 (94.0%)	13/217 (6.0%)	40/42 (95.2%)	2/42 (4.8%)	12/12 (100%)	0/12 (0.0%)	4/5 (80.0%)	1/5 (20.0%)	120/125 (96.0%)	5/125 (4.0%)	23/25 (92.0%)	2/25 (8.0%)
	**Negative**	566/1448 (39.1%)	882/1448 (60.9%)	467/1623 (28.8%)	1156/1623 (71.2%)	426/1589 (26.8%)	1163/1589 (73.2%)	330/1595 (20.7%)	1265/1595 (79.3%)	313/1413 (22.2%)	1100/1413 (77.8%)	220/1513 (14.5%)	1293/1513 (85.5%)
	**Sensitivity**	26.5%		7.9%		2.7%		1.2%		27.7%		9.5%	
	**Specificity**	98.5%		99.8%		100%		99.9%		99.5%		99.8%	

[a], positive by qPCR indicates sample was positive for *Shigella*-EIEC

[b], positive by stool culture indicates sample had *Shigella* cultured and identified by classical microbiological methods
