# Supplementary material for: Shigella in Africa: New Insights From the Vaccine Impact on Diarrhea in Africa (VIDA) Study
Source: Clin Infect Dis. 2023 Apr 19;76(Suppl 1):S66–76. doi: 10.1093/cid/ciac969 (PMC10116563; doi:10.1093/cid/ciac969)
Supplement: ciac969_Supplementary_Data [file ciac969_supplementary_data.zip › Supplementary table_2.pdf]

Supplementary Table 2. Number and percent of children with bloody versus watery diarrhea at each site by age-group.

	Age (months)		
	**0-11**	**12-23**	**24-59**
**All Sites**	N = 129	N = 493	N = 335
Bloody	51 (39.5%)	200 (40.6%)	168 (50.1%)
Acute watery	72 (55.8%)	252 (51.1%)	155 (46.3%)
Persistent watery	6 (4.7%)	41 (8.3%)	12 (3.6%)
**The Gambia**	N = 78	N = 259	N = 180
Bloody	38 (48.7%)	153 (59.1%)	108 (60.0%)
Acute watery	39 (50.0%)	90 (34.7%)	67 (37.2%)
Persistent watery	1 (1.3%)	16 (6.2%)	5 (2.8%)
**Mali**	N = 13	N = 89	N = 48
Bloody	2 (15.4%)	12 (13.5%)	11 (22.9%)
Acute watery	10 (76.9%)	76 (85.4%)	36 (75.0%)
Persistent watery	1 (7.7%)	1 (1.1%)	1 (2.1%)
**Kenya**	N = 38	N = 145	N = 107
Bloody	11 (28.9%)	35 (24.1%)	49 (45.8%)
Acute watery	23 (60.5%)	86 (59.3%)	52 (48.6%)
Persistent watery	4 (10.5%)	24 (16.6%)	6 (5.6%)
