# Supplementary material for: Shigella in Africa: New Insights From the Vaccine Impact on Diarrhea in Africa (VIDA) Study
Source: Clin Infect Dis. 2023 Apr 19;76(Suppl 1):S66–76. doi: 10.1093/cid/ciac969 (PMC10116563; doi:10.1093/cid/ciac969)
Supplement: ciac969_Supplementary_Data [file ciac969_supplementary_data.zip › Supplementary table_3.pdf]

Supplementary Table 3. Clinical syndromes among 0-to 11-month-old MSD cases with watery diarrhea attributable to *Shigella* alone versus 0-to 11-month-old MSD cases with watery diarrhea attributable to a pathogen other than *Shigella*.

		Acute or persistent watery		
		Shigella **N=48**	**Any other pathogen N=734**	**P-value**
Vesikari score	Mild	15 (31.2%)	119 (16.2%)	0.0016
	Moderate	23 (47.9%)	286 (39.0%)	
	Severe	10 (20.8%)	329 (44.8%)	
WHO dehydration	No dehydration	3 (6.2%)	38 (5.2%)	0.8435
	Some dehydration	38 (79.2%)	573 (78.1%)	
	Severe dehydration	7 (14.6%)	123 (16.8%)	
Mental status	Normal	37 (77.1%)	495 (67.4%)	0.4080
	Restless, irritable	9 (18.8%)	198 (27.0%)	
	Lethargic/unconscious	2 (4.2%)	41 (5.6%)	
Belly pain/abdominal cramps	Yes	26 (54.2%)	335 (45.6%)	0.1161
Axillary temperature >38°C or parental perception	Yes	28 (58.3%)	449 (61.2%)	0.7750
Skin	Normal	34 (70.8%)	463 (63.1%)	0.6122
	Slow return	14 (29.2%)	263 (35.8%)	
	Very slow return	0 (0.0%)	8 (1.1%)	
Sunken eyes	Yes	48 (100%)	725 (98.8%)	1.0000
Very thirsty	Yes	40 (83.3%)	633 (86.2%)	0.5987
Mouth	Normal	13 (27.1%)	122 (16.6%)	0.1332
	Somewhat dry	34 (70.8%)	566 (77.1%)	
	Very dry	1 (2.1%)	46 (6.3%)	
Vomiting (Any)	Yes	18 (37.5%)	495 (67.4%)	<0.0001
Max # of vomiting episodes in one day	1	5 (27.8%)	66 (13.3%)	0.1993
	2-4	12 (66.7%)	357 (72.1%)	
	5 or more	1 (5.6%)	72 (14.5%)	
Admitted to hospital	Yes	3 (6.2%)	52 (7.1%)	1.0000
IV dehydration administered/prescribed	Yes	4 (8.3%)	73 (9.9%)	1.0000

		Acute or persistent watery		
		Shigella **N=48**	**Any other pathogen N=734**	**P-value**
Days of diarrhea (for V.S.)	1-4 days	46 (95.8%)	643 (87.6%)	0.2869
	5 days	2 (4.2%)	61 (8.3%)	
	>=6 days	0 (0.0%)	30 (4.1%)	
Max # of loose stools in one day	1-3 in a day	9 (18.8%)	139 (18.9%)	0.4399
	4-5 in a day	26 (54.2%)	451 (61.4%)	
	>=6 in a day	13 (27.1%)	144 (19.6%)	
Rectal straining	Yes	7 (14.6%)	138 (18.8%)	0.5942
Cough	Yes	21 (43.8%)	345 (47.0%)	0.7731
Difficulty breathing	Yes	3 (6.2%)	61 (8.3%)	0.7895
Change in HAZ	Median (IQR)	-0.29 (-0.57, -0.10)	-0.38 (-0.64, -0.06)	0.5050
Duration of diarrhea (days)	Median (IQR)	6.5 (4, 10)	5 (4, 8)	0.3233
Duration of vomiting (days)	Median (IQR)	2 (1, 2.75)	2 (2, 3)	0.3915

Shigella defined as an AF >= 0.5 and no other tested pathogens with an AF >= 0.5. Any other pathogen was defined as *Shigella* AF=0 and AF>=0.5 for any other pathogen.
P-values from Wilcoxon rank sum test for continuous and Chi square or Fisher’s exact test for categorical variables.
