# Supplementary material for: Shigella in Africa: New Insights From the Vaccine Impact on Diarrhea in Africa (VIDA) Study
Source: Clin Infect Dis. 2023 Apr 19;76(Suppl 1):S66–76. doi: 10.1093/cid/ciac969 (PMC10116563; doi:10.1093/cid/ciac969)
Supplement: ciac969_Supplementary_Data [file ciac969_supplementary_data.zip › Supplementary table_4.pdf]

Supplementary Table 4. Clinical syndromes among 12-to 23-month-old MSD cases with watery diarrhea attributable to *Shigella* alone versus 12-to 23-month-old MSD cases with watery diarrhea attributable to a pathogen other than *Shigella*.

		Acute or persistent watery		
		**Shigella N=161**	**Any other pathogen N=584**	**P-value**
Vesikari score	Mild	50 (31.1%)	118 (20.2%)	0.0001
	Moderate	73 (45.3%)	225 (38.5%)	
	Severe	38 (23.6%)	240 (41.1%)	
WHO dehydration	No dehydration	10 (6.2%)	17 (2.9%)	0.0786
	Some dehydration	120 (74.5%)	472 (80.8%)	
	Severe dehydration	31 (19.3%)	95 (16.3%)	
Mental status	Normal	112 (69.6%)	377 (64.6%)	0.0826
	Restless, irritable	39 (24.2%)	186 (31.8%)	
	Lethargic/unconscious	10 (6.2%)	21 (3.6%)	
Belly pain/abdominal cramps	Yes	73 (45.3%)	255 (43.7%)	0.7401
Axillary temperature >38°C or parental perception	Yes	104 (64.6%)	356 (61.0%)	0.4537
Skin	Normal	101 (62.7%)	396 (67.8%)	0.4283
	Slow return	59 (36.6%)	182 (31.2%)	
	Very slow return	1 (0.6%)	6 (1.0%)	
Sunken eyes	Yes	154 (95.7%)	570 (97.6%)	0.1848
Not able to drink/drink poorly	Yes	2 (1.2%)	6 (1.0%)	0.6850
Very thirsty	Yes	144 (89.4%)	533 (91.3%)	0.5597
Mouth	Normal	34 (21.1%)	94 (16.1%)	0.0795
	Somewhat dry	123 (76.4%)	454 (77.7%)	
	Very dry	4 (2.5%)	36 (6.2%)	
Vomiting (Any)	Yes	62 (38.5%)	374 (64.0%)	<0.0001
Max # of vomiting episodes in one day	1	13 (21.0%)	41 (11.0%)	0.0135
	2-4	45 (72.6%)	266 (71.1%)	
	5 or more	4 (6.5%)	67 (17.9%)	
Admitted to hospital	Yes	11 (6.8%)	33 (5.7%)	0.6653
IV dehydration administered/prescribed	Yes	12 (7.5%)	51 (8.7%)	0.7214
Days of diarrhea (for V.S.)	1-4 days	141 (87.6%)	526 (90.1%)	0.1498
	5 days	13 (8.1%)	48 (8.2%)	
	>=6 days	7 (4.3%)	10 (1.7%)	

		Acute or persistent watery		
		**Shigella N=161**	**Any other pathogen N=584**	**P-value**
Max # of loose stools in one day	1-3 in a day	35 (21.7%)	118 (20.2%)	0.4911
	4-5 in a day	89 (55.3%)	352 (60.3%)	
	>=6 in a day	37 (23.0%)	114 (19.5%)	
Rectal straining	Yes	28 (17.4%)	82 (14.0%)	0.4636
Rectal prolapse	Yes	1 (0.6%)	5 (0.9%)	1.0000
Cough	Yes	74 (46.0%)	251 (43.0%)	0.5578
Difficulty breathing	Yes	11 (6.8%)	32 (5.5%)	0.6449
Change in HAZ	Median (IQR)	-0.23 (-0.38, 0.03)	-0.26 (-0.45, -0.01)	0.0441
Duration of vomiting (days)	Median (IQR)	2 (1, 3)	2 (2, 3)	0.1793
Convulsion	Yes	0 (0.0%)	5 (0.9%)	0.5907
Duration of diarrhea (days)	Median (IQR)	6 (4, 10)	5 (4, 8)	0.0599

Shigella defined as an AF >= 0.5 and no other tested pathogens with an AF >= 0.5. Any other pathogen was defined as *Shigella* AF=0 and AF>=0.5 for any other pathogen.
P-values from Wilcoxon rank sum test for continuous and Chi square or Fisher's exact test for categorical variables.
