# Supplementary material for: Shigella in Africa: New Insights From the Vaccine Impact on Diarrhea in Africa (VIDA) Study
Source: Clin Infect Dis. 2023 Apr 19;76(Suppl 1):S66–76. doi: 10.1093/cid/ciac969 (PMC10116563; doi:10.1093/cid/ciac969)
Supplement: ciac969_Supplementary_Data [file ciac969_supplementary_data.zip › Supplementary table_5.pdf]

Supplementary Table 5. Clinical syndromes among 24-to 59-month-old MSD cases with watery diarrhea attributable to *Shigella* alone versus 24-to 59-month-old MSD cases with watery diarrhea attributable to a pathogen other than *Shigella*.

		**Acute or persistent watery**		
		**Shigella N=106**	**Any other pathogen N=363**	**P-value**
Vesikari score	Mild	28 (26.4%)	108 (29.8%)	0.0297
	Moderate	56 (52.8%)	142 (39.1%)	
	Severe	22 (20.8%)	113 (31.1%)	
WHO dehydration	No dehydration	6 (5.7%)	14 (3.9%)	0.6261
	Some dehydration	85 (80.2%)	299 (82.4%)	
	Severe dehydration	15 (14.2%)	50 (13.8%)	
Mental status	Normal	80 (75.5%)	262 (72.2%)	0.4394
	Restless, irritable	20 (18.9%)	87 (24.0%)	
	Lethargic/unconscious	6 (5.7%)	14 (3.9%)	
Belly pain/abdominal cramps	Yes	57 (53.8%)	193 (53.2%)	0.3476
Axillary temperature >38°C or parental perception	Yes	75 (70.8%)	208 (57.3%)	0.0038
Skin	Normal	79 (74.5%)	246 (67.8%)	0.3570
	Slow return	26 (24.5%)	113 (31.1%)	
	Very slow return	1 (0.9%)	4 (1.1%)	
Sunken eyes	Yes	103 (97.2%)	349 (96.1%)	0.7736
Not able to drink/drink poorly	Yes	4 (3.8%)	3 (0.8%)	0.0492
Very thirsty	Yes	98 (92.5%)	332 (91.5%)	0.8435
Mouth	Normal	24 (22.6%)	59 (16.3%)	0.0570
	Somewhat dry	80 (75.5%)	278 (76.6%)	
	Very dry	2 (1.9%)	26 (7.2%)	
Vomiting (Any)	Yes	42 (39.6%)	204 (56.2%)	0.0038
Max # of vomiting episodes in one day	1	13 (31.0%)	34 (16.7%)	0.0517
	2-4	25 (59.5%)	129 (63.2%)	
	5 or more	4 (9.5%)	41 (20.1%)	
Admitted to hospital	Yes	7 (6.6%)	30 (8.3%)	0.7239
IV dehydration administered/prescribed	Yes	9 (8.5%)	42 (11.6%)	0.4723
Days of diarrhea (for V.S.)	1-4 days	98 (92.5%)	338 (93.1%)	0.7887
	5 days	6 (5.7%)	15 (4.1%)	
	>=6 days	2 (1.9%)	10 (2.8%)	

		Acute or persistent watery		
		**Shigella N=106**	**Any other pathogen N=363**	**P-value**
Max # of loose stools in one day	1-3 in a day	20 (18.9%)	84 (23.1%)	0.6216
	4-5 in a day	65 (61.3%)	215 (59.2%)	
	>=6 in a day	21 (19.8%)	64 (17.6%)	
Rectal straining	Yes	14 (13.2%)	40 (11.0%)	0.6541
Rectal prolapse	Yes	0 (0.0%)	5 (1.4%)	0.5925
Cough	Yes	47 (44.3%)	156 (43.0%)	0.8902
Difficulty breathing	Yes	9 (8.5%)	27 (7.4%)	0.8802
Duration of vomiting (days)	Median (IQR)	1 (1, 2)	2 (1, 2)	0.0091
Convulsion	Yes	0 (0.0%)	2 (0.6%)	1.0000
Duration of diarrhea (days)	Median (IQR)	5 (3, 8)	4 (3, 7)	0.5493
Change in HAZ	Median (IQR)	-0.12 (-0.23, 0.03)	-0.11 (-0.24, 0.04)	0.7302

Shigella defined as AF >= 0.5 and no other pathogens with AF >= 0.5. Any other pathogen defined as Shigella AF=0 and AF>=0.5 for any other pathogen.
P-values from Wilcoxon rank-sum test for continuous and ordinal variables and Fisher's exact test for binary variables.
