# Supplementary material for: Shigella in Africa: New Insights From the Vaccine Impact on Diarrhea in Africa (VIDA) Study
Source: Clin Infect Dis. 2023 Apr 19;76(Suppl 1):S66–76. doi: 10.1093/cid/ciac969 (PMC10116563; doi:10.1093/cid/ciac969)
Supplement: ciac969_Supplementary_Data [file ciac969_supplementary_data.zip › Supplementary table_6.pdf]

Supplementary Table 6. Differences in clinical severity between *S. flexneri* and *S. sonnei.*

Characteristic	Value	*Shigella* spp.		P-value
		S. flexneri N=234	*S. sonnei* N=63	
Bloody diarrhea	Yes	141 (60.3%)	28 (44.4%)	0.0352
Persistent diarrhea (>=14 days)	Yes	20 (8.5%)	10 (15.9%)	0.1396
Fever	Yes	175 (74.8%)	50 (79.4%)	0.5571
Admitted to hospital	Yes	21 (9.0%)	1 (1.6%)	0.0557
Vomiting (Any)	Yes	91 (38.9%)	22 (34.9%)	0.6674
Max # of vomiting episodes in one day	1	19 (20.9%)	6 (27.3%)	0.4192
	2-4	65 (71.4%)	16 (72.7%)	
	5 or more	7 (7.7%)	0 (0.0%)	
WHO dehydration	No dehydration	48 (20.5%)	10 (15.9%)	0.2297
	Some dehydration	140 (59.8%)	45 (71.4%)	
	Severe dehydration	47 (19.7%)	8 (12.7%)	
Vesikari score	Mild	70 (29.9%)	23 (36.5%)	0.4704
	Moderate	109 (46.6%)	29 (46.0%)	
	Severe	55 (23.5%)	11 (17.5%)	
Vesikari score (integer)	Median (IQR)	8 (6, 10)	7 (6, 10)	0.2172
Duration of diarrhea (days)	Median (IQR)	5 (4, 8)	6 (3, 8.5)	0.8490
Max # of loose stools in one day	1-3 in a day	24 (10.3%)	8 (12.7%)	0.7882
	4-5 in a day	135 (57.7%)	37 (58.7%)	
	>= 6 in a day	75 (32.1%)	18 (28.6%)	

Cases with multiple *Shigella* serogroups detected were excluded (N=12).
P-values from Wilcoxon rank sum test for quantitative variables and chi square or Fisher's exact test for categorical variables.
