# Supplementary material for: Shigella in Africa: New Insights From the Vaccine Impact on Diarrhea in Africa (VIDA) Study
Source: Clin Infect Dis. 2023 Apr 19;76(Suppl 1):S66–76. doi: 10.1093/cid/ciac969 (PMC10116563; doi:10.1093/cid/ciac969)
Supplement: ciac969_Supplementary_Data [file ciac969_supplementary_data.zip › Supplementary table_7.pdf]

Supplementary Table 7. Proportion of each serogroup isolated from *Shigella*-positive children stratified by bloody versus watery diarrhea.

	The Gambia (N=207)		Mali (N=12)		Kenya (N=128)	
	Bloody	Watery*	Bloody	Watery	Bloody	Watery
S. boydii	14 (6.8%)	16 (7.7%)	0	1 (8.3%)	3 (2.3%)	7 (5.5%)
S. sonnei	15 (7.2%)	9 (4.3%)	1 (8.3%)	3 (25.0%)	12 (9.4%)	23 (18.0%)
S. dysenteriae	2 (1.0%)	4 (1.9%)	0	0	2 (1.6%)	0
S. flexneri	96 (46.4%)	50 (24.2%)	3 (25.0%)	4 (33.3%)	42 (32.8%)	39 (30.5%)

* An additional case of watery diarrhea in The Gambia had *Shigella* spp. (untypable) isolated.
